# TyHGB and CVD high-risk stratification: nonlinear association and discrimination in the ChinaHEART Luohe study

**DOI:** 10.3389/fendo.2026.1818472

**Published:** 2026-05-07

**Authors:** Zhiwei Huang, Jirui Cai, Jing Bai, Li Wu, Yabo Huang, Li Guo, Jin Wang, Guirang Zhao, Qiaotao Xie, Haoran Wang

**Affiliations:** 1 Luohe Central Hospital, Luohe Medical College, Luohe, China; 2 Henan University College of Medicine, Henan University, Kaifeng, China; 3 Luohe Center for Disease Control and Prevention, Luohe, China; 4 Henan Key Laboratory of Fertility Protection and Aristogenesis, Luohe, China

**Keywords:** cardiovascular disease high-risk, ChinaHEART cohort, risk stratification, triglyceride-glucose index (TyG), TyHGB, WHO CVD risk charts

## Abstract

**Introduction:**

TyHGB is a composite metabolic index integrating triglyceride-to-high-density lipoprotein cholesterol ratio, fasting plasma glucose, and body mass index, but its association with World Health Organization (WHO)-defined cardiovascular disease (CVD) high-risk status in community settings remains unclear.

**Methods:**

We analyzed 6,755 adults from a community-based ChinaHEART Luohe screening cohort (2021–2022). CVD high-risk was defined as an estimated 10-year WHO CVD risk ≥20%. TyHGB was examined per 1-unit increase and as high versus low using a receiver operating characteristic (ROC)-derived cutoff (Youden index; approximately 7.6). Logistic regression and restricted cubic splines were used to assess associations and potential nonlinearity. Discrimination was evaluated using ROC curves with DeLong’s tests, and incremental value beyond conventional risk factors was assessed using integrated discrimination improvement (IDI) and net reclassification improvement (NRI).

**Results:**

The prevalence of CVD high-risk was 22%. TyHGB was independently associated with CVD high-risk (fully adjusted OR 1.131 per 1-unit increase), and high TyHGB (≥7.6) was associated with higher odds (OR 1.642). In unadjusted analyses, TyHGB showed higher discrimination than TyG (AUC 0.624 vs 0.585). Adding TyHGB to the base model modestly improved reclassification (IDI 0.003; NRI 0.216). TyHGB was also retained in exploratory machine-learning analyses.

**Discussion:**

TyHGB was positively associated with WHO-defined CVD high-risk and provided modest incremental discrimination and reclassification for cross-sectional high-risk classification beyond conventional risk factors, supported by sensitivity analyses. These findings relate to estimated risk status rather than adjudicated incident CVD events.

## Introduction

Cardiovascular disease (CVD) remains a leading cause of morbidity and mortality worldwide, and identifying individuals at high risk is central to efficient prevention strategies (1). Risk prediction tools, including the World Health Organization (WHO) CVD risk chart, enable population-level screening and prioritization of preventive interventions (2). However, conventional risk factors do not fully capture residual risk, motivating interest in pragmatic metabolic indices derived from routine measurements (3, 4).

The triglyceride-glucose (TyG) index, calculated from fasting triglycerides and glucose, has emerged as a convenient surrogate for insulin resistance and has been associated with cardiometabolic outcomes in diverse populations (5–11). Nonetheless, TyG alone may not adequately reflect the combined metabolic burden contributed by adiposity and protective lipid fractions. Composite indices combining routine metabolic and anthropometric measures may offer additional value for CVD risk stratification (12–14).

TyHGB is a composite metabolic index integrating ratio of triglycerides and high-density lipoprotein cholesterol (TG/HDL-C), fasting plasma glucose (FPG), and BMI information (as available in the ChinaHEART baseline dataset), potentially capturing both glyco-lipid perturbation and adiposity-related risk. Yet evidence regarding the association between TyHGB and clinically meaningful high-risk categories at the community level remains limited.

Using a community-based branch of the ChinaHEART cohort in Luohe, China (15, 16), we aimed to (1) evaluate the association of TyHGB with CVD high-risk status defined by WHO CVD risk charts, (2) explore potential nonlinear dose–response patterns using restricted cubic splines, (3) compare discrimination performance between TyHGB and traditional TyG, and (4) assess incremental discrimination and reclassification performance of adding TyHGB versus TyG beyond a base risk-factor model. TyG was additionally used as a sensitivity analysis to examine robustness.

## Methods

### Study design and population

This cross-sectional study was conducted in accordance with the Strengthening the Reporting of Observational Studies in Epidemiology (STROBE) statement and the Declaration of Helsinki (17, 18). It was based on a branch of the ChinaHEART cohort (15, 16), a government-supported public health initiative aimed at identifying CVD risk and implementing targeted community interventions nationwide. Community residents were recruited from township hospitals in Luohe, Henan Province, China, between March 2021 and February 2022. Eligible participants were aged 35–75 years, were permanent residents of the project site (≥6 months residence in the preceding 12 months), and provided written informed consent. The study protocol was approved by the institutional ethics committee of Fuwai Hospital and filed with the local ethics committee of Luohe Central Hospital.

A total of 6,860 participants were enrolled. For descriptive and TyHGB-based analyses, 105 participants (1.5%) with missing TyHGB were excluded, leaving 6,755 individuals. For logistic regression analyses directly comparing TyHGB and TyG under identical covariate adjustment, we used a shared complete-case dataset requiring complete data on the outcome, both exposure variables, and all prespecified covariates, resulting in 6,750 participants.

### Data collection and measurements

Trained staff conducted face-to-face interviews using standardized questionnaires to collect demographic information (e.g., age, sex), lifestyle factors (current smoking, alcohol consumption), self-reported comorbidities and medical history, and current medication use. Physical measurements included height, weight, and waist circumference; BMI was calculated as weight (kg) divided by height squared (m^2^). Blood pressure was measured using an electronic sphygmomanometer after at least 5 minutes of rest in a seated position, with two readings recorded. Heart rate was also recorded. Laboratory measurements included fasting glucose and lipid profiles (including HDL-C, triglycerides, and non-HDL-C when available) obtained via standard procedures implemented in the ChinaHEART community screening workflow.

### Exposure definitions: TyHGB and TyG

TyHGB was the primary exposure. TyHGB is a composite metabolic index integrating triglycerides (TG), high-density lipoprotein cholesterol (HDL-C), fasting plasma glucose (FPG), and BMI to reflect combined glyco-lipid perturbation and adiposity burden. TyHGB was calculated using the original published formula reported by Song et al. (19): TyHGB=(TG/HDL-C)+0.7×FPG+0.1×BMI. In the present study, we applied this published formula directly and did not re-derive or re-optimize its coefficients. A subsequent study applying the same index was also cited for context (20). The TyG index was calculated as TyG =ln([TG×FPG]/2), as previously described (5–7). where TG denotes fasting triglycerides and FPG denotes fasting plasma glucose.

In this study, Glucose and lipid measurements were recorded in mmol/L. No unit conversion was performed for TyHGB; TyHGB was calculated using TG/HDL-C and FPG in mmol/L (and BMI in kg/m^2^), consistent with the originally reported formula (19). In contrast, for TyG, because the standard formula is defined as ln[TG (mg/dL) × FPG (mg/dL)/2], TG and FPG were converted from mmol/L to mg/dL before calculation (TG × 88.57; FPG × 18) (21). TyHGB was analyzed (1) as a continuous variable (per 1-unit increase) and (2) as a categorical variable (high vs low) using a receiver operating characteristic (ROC)-derived cutoff (Youden index; approximately 7.6). TyG was analyzed in parallel as a continuous variable (per 1-unit increase), and its nonlinear association and discriminatory performance were assessed as sensitivity analyses.

### Outcome definition: CVD high-risk status

CVD high-risk status was defined using WHO CVD risk chart (2). Based on the estimated 10-year risk of CVD, individuals with a risk ≥20% were classified as CVD high-risk, and the remainder were classified as non-high risk.

### Covariates

Covariates were selected *a priori* based on clinical relevance and data availability in the baseline dataset. Stepwise logistic regression models were constructed as follows:

Model 1: crude model including exposure only.

Model 2: adjusted for age and sex.

Model 3: additionally adjusted for current smoking, alcohol consumption, waist circumference, systolic blood pressure, family history of stroke, and heart rate.

### Statistical analysis

Baseline characteristics were summarized overall and by TyHGB categories (low vs high). Continuous variables were presented as median (quartile) and compared using the Mann-Whitney U test. Categorical variables were presented as counts (percentages) and compared using Pearson’s chi-squared test.

Associations between TyHGB/TyG and CVD high-risk status were evaluated using logistic regression with odds ratios (ORs) and 95% confidence intervals (CIs). To improve interpretability on an absolute scale, we additionally estimated adjusted absolute differences (percentage-point differences) in WHO-defined CVD high-risk status using marginal standardization based on the same logistic regression models. Potential nonlinear relationships were explored using restricted cubic splines (RCS). Discriminatory performance was assessed using ROC curves with bootstrap area under the curve (AUC) estimates; differences between AUCs were tested using DeLong’s method. Incremental discrimination and reclassification performance beyond the base model (Model 3 covariates) was quantified using integrated discrimination improvement (IDI) and net reclassification improvement (NRI). As a sensitivity analysis to address potential covariate overlap with WHO risk-chart inputs, we additionally compared discrimination on the same analytic sample across four models: the full Model 3 plus TyHGB, a reduced model excluding systolic blood pressure, waist circumference, and current smoking plus TyHGB, the same reduced model plus TyG, and the reduced base model without either index, using bootstrap AUCs and paired DeLong tests. As a complementary supplementary analysis, we further evaluated the discrimination of TyHGB for self-reported history of cardiovascular disease, a non-chart-derived prevalent outcome, under the same Model 1–3 framework using bootstrap AUCs and paired DeLong tests. Subgroup analyses were conducted using unadjusted models within strata to avoid overadjustment and sparse-data instability; interaction was tested via likelihood ratio test (LRT). To account for multiple testing in subgroup interaction analyses, Benjamini-Hochberg false discovery rate (FDR) adjustment was additionally applied separately within the continuous TyHGB interaction family and the categorical TyHGB interaction family. All analyses were conducted using R software (version 4.5.2), and statistical significance was defined as a two-sided *P* < 0.05. Missing-data handling was analysis-specific. Baseline and TyHGB-based descriptive analyses used participants with available TyHGB data, whereas the regression analyses used the shared complete-case dataset described above to ensure direct comparison of TyHGB and TyG under identical covariate adjustment. As a sensitivity analysis, we performed multiple imputation by chained equations (MICE) under a missing-at-random assumption, using 20 imputations and 20 iterations. The outcome was not imputed; TyHGB categories were re-derived within each imputed dataset, and pooled estimates were obtained using Rubin’s rules.

### Machine learning-based feature selection and model interpretability (LASSO+RF+SHAP)

To provide complementary evidence for the relative importance of TyHGB among candidate predictors, we conducted an exploratory machine learning analysis. Candidate variables were pre-specified as age, sex, LDL-C, non–HDL-C, fasting glucose, triglycerides, current smoking, alcohol use, history of hypertension, history of diabetes mellitus, and TyHGB. Categorical variables were one-hot encoded; continuous variables were standardized. Missing continuous values were imputed using the median, and missing categorical values were treated as a separate category.

**Figure 1:**
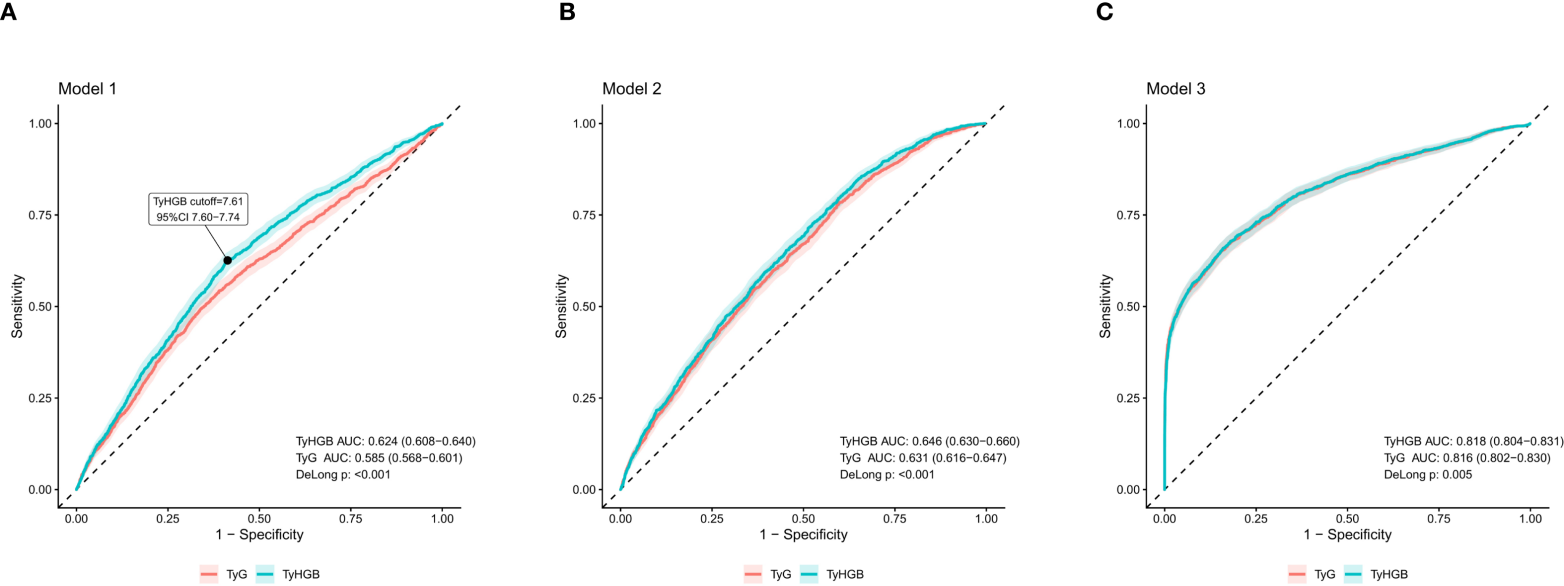
Comparative discrimination of TyHGB versus TyG for WHO-defined CVD high-risk status. Receiver operating characteristic (ROC) curves are shown for TyHGB and TyG under **(A)** Model 1 (crude), **(B)** Model 2 (partially adjusted), and **(C)** Model 3 (fully adjusted). Areas under the curve (AUCs) are presented with bootstrap 95% CIs, and between-index differences were tested using DeLong’s method. The ROC/Youden-derived cutoff for TyHGB (7.61; 95% CI 7.60–7.74) is indicated.

We applied least absolute shrinkage and selection operator (LASSO) logistic regression with 10-fold cross-validation and selected the penalty parameter at λ1se. Variables with non-zero coefficients were retained. A random forest classifier was then trained using the LASSO-selected variables (70/30 train–test split; 500 trees), and discrimination was evaluated using the area under the ROC curve (AUC). To interpret model predictions, Shapley additive explanations (SHAP) values were computed (fastshap; nsim = 30) and visualized using a beeswarm plot, where positive SHAP values indicate increased predicted probability of CVD high-risk (**Figure 1**). This analysis was exploratory and intended to support variable importance rather than to develop a replacement for the WHO CVD risk chart. In the SHAP summary (beeswarm) plot, features are ordered by global importance quantified as the mean absolute SHAP value across individuals. Therefore, “rank x of y selected” indicates that the feature is the x-th most influential variable among the y predictors retained after LASSO selection and used in the random forest model.

## Results

### Study population

A total of 6,860 community-dwelling adults were enrolled. After excluding 105 participants with missing TyHGB, 6,755 individuals remained for the overall TyHGB-based analytic sample. For the shared logistic regression dataset, 6,750 participants had complete data on the outcome, TyHGB, TyG, and all prespecified covariates, whereas 110 participants (1.6%) were excluded because of missingness in at least one required regression variable. Within this regression variable set, missingness involved 57 participants with concurrent missing TyHGB and TyG, 48 with missing TyHGB only, and 5 with missing heart rate (**Supplementary Table 2**).

### ROC-derived cutoff for TyHGB (Youden index) and group definition

Receiver operating characteristic (ROC) analysis was performed to determine the optimal TyHGB threshold for discriminating CVD high-risk status (**Figure 1**). Using the Youden index, the optimal cutoff for TyHGB was 7.61 (95% CI 7.60–7.74). Participants were therefore categorized into a low TyHGB group (<7.6) and a high TyHGB group (≥7.6) for subsequent baseline comparisons and categorical regression analyses. The cutoff was rounded to one decimal place for grouping and presentation.

### Baseline characteristics by TyHGB category

Overall, the median age was 58 (51, 66) years, and 2,562 (38%) participants were men (**Table 1**). Compared with the low TyHGB group (N = 3,622), the high TyHGB group (N = 3,133) showed higher adiposity and hemodynamic burden, including higher weight, waist circumference, BMI, systolic blood pressure (SBP), diastolic blood pressure (DBP), and heart rate (all *P* < 0.001). The high TyHGB group also exhibited a less favorable metabolic profile, with lower HDL-C and higher non-HDL-C, triglycerides, and fasting glucose (all *P* < 0.001). The prevalence of CVD high-risk status was 1,468/6,755 (22%) overall and was substantially higher in the high TyHGB group than in the low TyHGB group (29% vs 15%, *P* < 0.001).

**Table 1:** Baseline characteristics.

Variable	Overall N = 6,755^1^	Low TyHGB N = 3,622	High TyHGB N = 3,133	*P*-value
Age (years)	58 (51, 66)	58 (49, 67)	59 (52, 66)	<0.001
Sex (male)	2,562 (38%)	1,512 (42%)	1,050 (34%)	<0.001
Current smoking	1,357 (20%)	808 (22%)	549 (18%)	<0.001
Alcohol consumption	370 (5.5%)	153 (4.2%)	217 (6.9%)	<0.001
Height (cm)	159 (154, 166)	160 (155, 167)	158 (153, 164)	<0.001
Weight (kg)	64 (58, 72)	61 (55, 68)	68 (61, 75)	<0.001
Waist circumference (cm)	86 (80, 92)	83 (78, 88)	90 (84, 97)	<0.001
Systolic blood pressure (mmHg)	137 (126, 150)	133 (123, 146)	143 (131, 152)	<0.001
Diastolic blood pressure (mmHg)	83 (77, 91)	81 (75, 88)	86 (78, 94)	<0.001
Heart rate (bpm)	75 (70, 83)	74 (69, 81)	77 (71, 84)	<0.001
Body mass index (kg/m^2^)	25.1 (23.0, 27.3)	23.7 (22.0, 25.5)	26.8 (24.8, 29.1)	<0.001
High-density lipoprotein cholesterol (mmol/L)	1.43 (1.23, 1.66)	1.51 (1.32, 1.75)	1.32 (1.15, 1.53)	<0.001
Non-high-density lipoprotein cholesterol (mmol/L)	3.31 (2.66, 3.98)	3.07 (2.49, 3.66)	3.63 (2.94, 4.38)	<0.001
Triglycerides (mmol/L)	1.52 (1.14, 2.03)	1.26 (1.00, 1.56)	2.00 (1.54, 2.74)	<0.001
Fasting glucose (mmol/L)	5.40 (5.19, 6.00)	5.20 (4.99, 5.50)	6.00 (5.40, 6.90)	<0.001
Cardiovascular disease high-risk status	1,468 (22%)	544 (15%)	924 (29%)	<0.001
Hypertension	1,552 (23%)	504 (14%)	1,048 (33%)	<0.001
Diabetes mellitus	423 (6.3%)	27 (0.7%)	396 (13%)	<0.001
Stroke	191 (2.8%)	92 (2.5%)	99 (3.2%)	0.145
Dyslipidemia	1,189 (18%)	393 (11%)	796 (25%)	<0.001
Family history of stroke	155 (2.3%)	62 (1.7%)	93 (3.0%)	<0.001
Antihypertensive medication	897 (13%)	276 (7.6%)	621 (20%)	<0.001
Antidiabetic medication	262 (3.9%)	20 (0.6%)	242 (7.7%)	<0.001
Statin use	204 (3.0%)	64 (1.8%)	140 (4.5%)	<0.001

Continuous variables: median (Q1, Q3). Dichotomous variables: n (%). Mann-Whitney U test; Pearson's Chi-squared test.

### Association of TyHGB with CVD high-risk status

In logistic regression analyses, TyHGB was positively associated with CVD high-risk status across all models (**Table 2**). When treated as a continuous variable, each 1-unit increase in TyHGB was associated with higher odds of CVD high-risk (Model 3: OR 1.131, 95% CI 1.081-1.183; *P* < 0.001). When analyzed categorically using the ROC-derived cutoff, participants with high TyHGB (≥7.6) had significantly higher odds of CVD high-risk compared with those with low TyHGB (Model 3: OR 1.642, 95% CI 1.408–1.916; *P* < 0.001). In the fully adjusted model, these associations corresponded to adjusted absolute differences of 1.6 percentage points per 1-unit increase in TyHGB and 6.2 percentage points for high versus low TyHGB in WHO-defined CVD high-risk classification (**Supplementary Table 1**). Results were materially unchanged in the missing-data sensitivity analysis using MICE (**Supplementary Table 2**). In the fully adjusted pooled models, the OR was 1.134 (95% CI 1.084–1.186) per 1-unit increase in TyHGB, 1.652 (95% CI 1.416–1.927) for high versus low TyHGB, and 1.361 (95% CI 1.188–1.559) per 1-unit increase in TyG, closely matching the corresponding shared complete-case estimates.

**Table 2:** Associations of TyHGB and TyG with CVD high-risk status.

Variable	Model 1		Model 2		Model 3	
	OR (95% CI)	*P*-value	OR (95% CI)	*P*-value	OR (95% CI)	*P*-value
TyHGB (per 1-unit increase)	1.260 (1.217, 1.306)	<0.001	1.263 (1.219, 1.309)	<0.001	1.131 (1.081, 1.183)	<0.001
High TyHGB (≥7.6) vs. Low TyHGB (<7.6)	2.359 (2.094, 2.658)	<0.001	2.398 (2.124, 2.707)	<0.001	1.642 (1.408, 1.916)	<0.001
TyG (per 1-unit increase)	1.777 (1.591, 1.984)	<0.001	1.915 (1.708, 2.147)	<0.001	1.343 (1.171, 1.540)	<0.001

Model 1: crude (exposure only). Model 2: adjusted for age and sex. Model 3: additionally adjusted for current smoking, alcohol consumption, waist circumference, systolic blood pressure, family history of stroke, and heart rate. N = 6750.

### Sensitivity analysis using TyG

TyG showed consistent associations in sensitivity analyses (**Table 2**). Per 1-unit increase in TyG, the fully adjusted odds of CVD high-risk increased (Model 3: OR 1.343, 95% CI 1.171–1.540; *P* < 0.001), supporting the robustness of the glyco-lipid metabolic signal captured by TyHGB.

### Dose–response patterns (restricted cubic spline)

**Figure 2:**
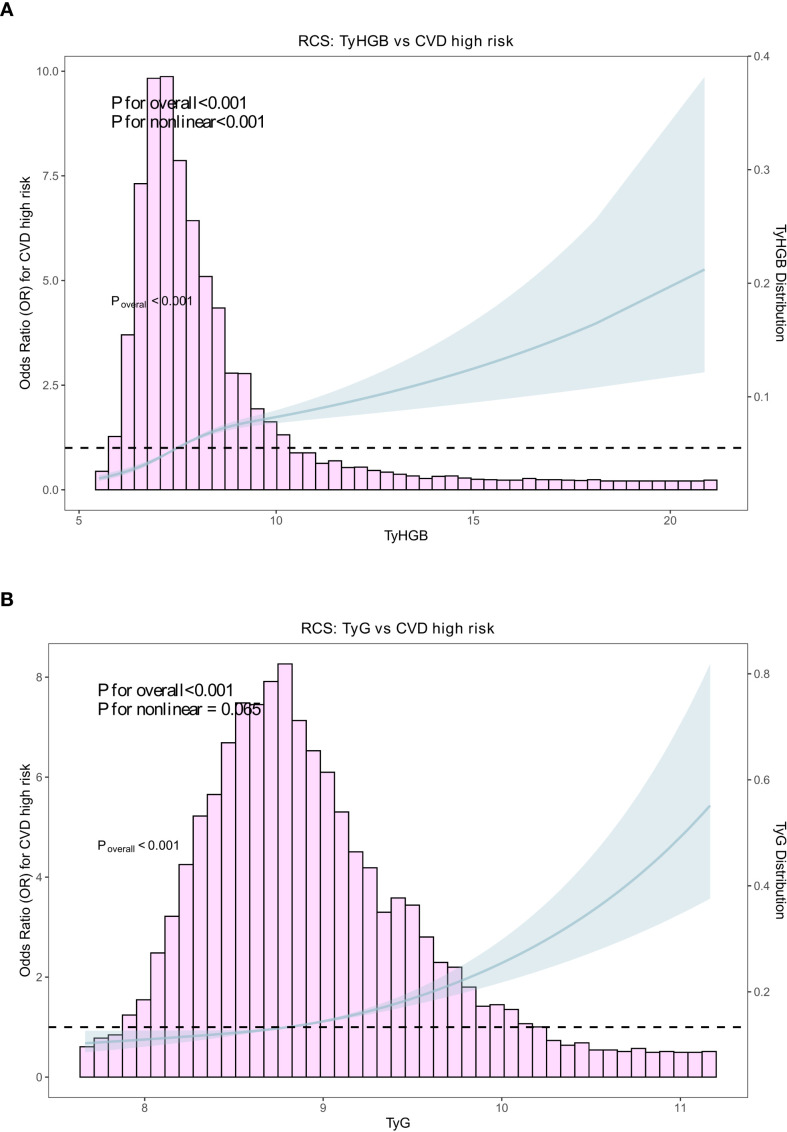
Restricted cubic spline analyses for the unadjusted dose–response association of TyHGB and TyG with WHO-defined CVD high-risk status. Unadjusted odds ratios (ORs) and 95% confidence intervals (CIs) are shown for **(A)** TyHGB and **(B)** TyG in relation to CVD high-risk status. The solid line represents the estimated OR and the shaded area indicates the 95% CI. Histograms display the distribution of TyHGB/TyG in the analytic sample. *P* values for overall association and nonlinearity are provided in each panel.

RCS analyses were performed using unadjusted models to visualize the dose-response pattern. Restricted cubic spline analyses suggested a significant nonlinear association between TyHGB and CVD high-risk status (*P* for nonlinearity <0.001; *P* for overall <0.001). For TyG, the overall association was significant, while evidence for nonlinearity was weaker (*P* for nonlinearity =0.065) (**Figure 2**).

### Distributional differences by outcome

**Figure 3:**
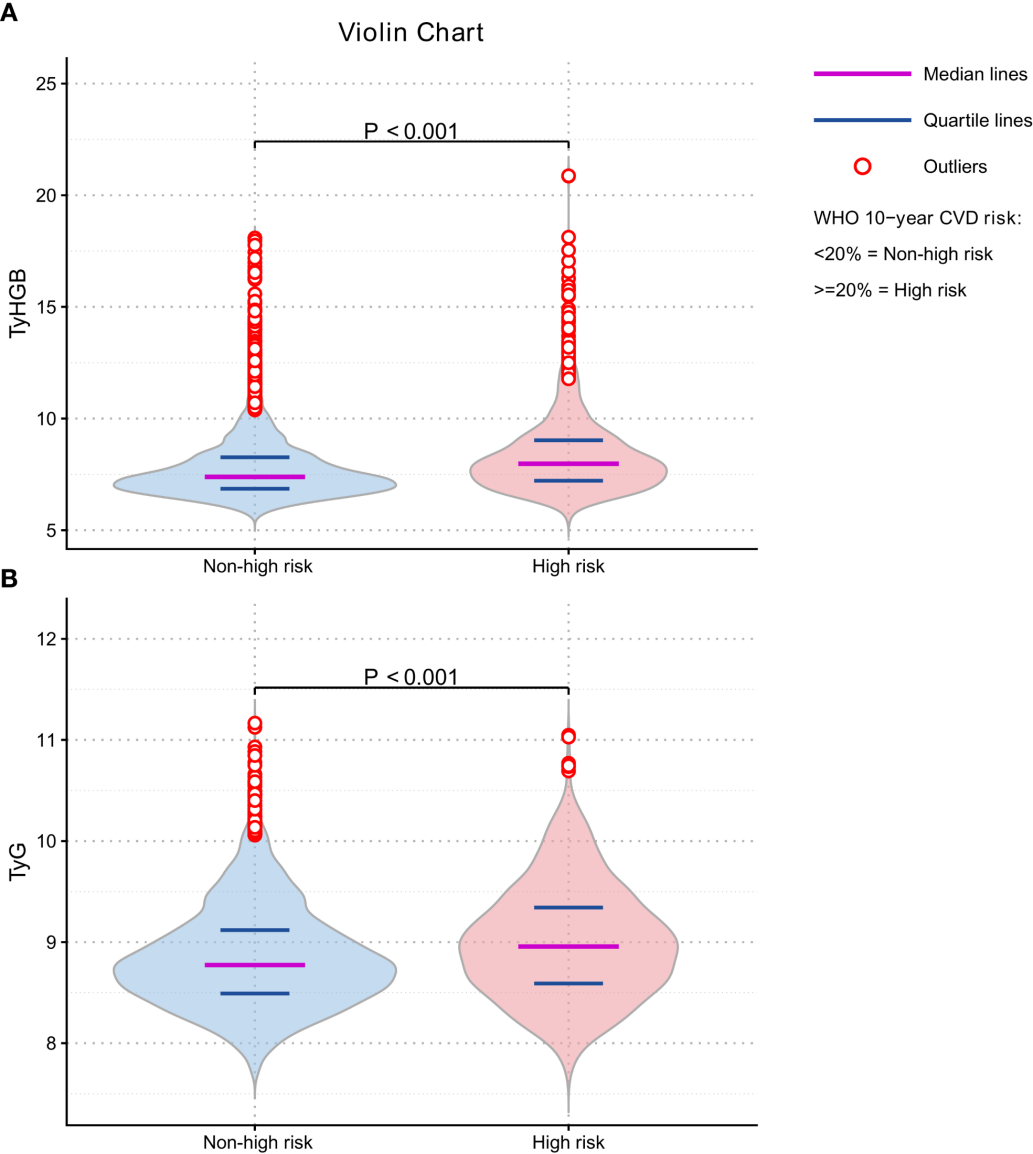
Distribution of TyHGB and TyG by WHO-defined CVD high-risk status. Violin plots (with embedded boxplots) depict the distributions of **(A)** TyHGB and **(B)** TyG in participants classified as non-high risk versus high risk (10-year WHO CVD risk ≥20%). Median and interquartile range are shown within each box. *P* values were derived from the Mann-Whitney U test.

Violin plots demonstrated clear distributional shifts for both indices: participants classified as CVD high-risk had higher TyHGB and higher TyG than those without CVD high-risk (both *P* < 0.001) (**Figure 3**).

### Discrimination performance and ROC comparison (TyHGB vs TyG)

In unadjusted comparisons, TyHGB showed higher discrimination than TyG (AUC 0.624 [0.608–0.640] vs 0.585 [0.568–0.601]; DeLong *P* < 0.001). In adjusted models, the AUC differences between TyHGB and TyG were statistically significant but numerically very small (e.g., Model 3: 0.818 vs 0.816; DeLong P = 0.005), indicating limited practical separation beyond conventional covariates (**Figure 1**). In an overlap-excluded sensitivity ROC analysis, removal of systolic blood pressure, waist circumference, and current smoking from the comparison framework did not change the overall direction of the findings: both reduced-model TyHGB and reduced-model TyG improved discrimination versus the reduced base model, and reduced-model TyHGB remained higher than reduced-model TyG (all DeLong *P* < 0.001; **Supplementary Figure 1A**). In a complementary supplementary ROC analysis using self-reported history of CVD as a non-chart-derived outcome, TyHGB-containing models yielded AUCs of 0.554 (0.521–0.586), 0.701 (0.674–0.729), and 0.731 (0.705–0.756) under Models 1, 2, and 3, respectively; all pairwise DeLong comparisons were significant (all *P* < 0.001; **Supplementary Figure 1B**).

### Incremental discrimination and reclassification performance (NRI and IDI)

Adding TyHGB to the fully adjusted base model improved discrimination and reclassification (Model 3+TyHGB: IDI 0.003 and NRI 0.216, both *P* < 0.0001). Adding TyG also improved prediction (IDI 0.002, NRI 0.143), whereas the direct comparison between “Model 3+TyG” and “Model 3+TyHGB” did not show significant differences in IDI or NRI (**Table 3**).

**Table 3:** Incremental discrimination and reclassification performance of TyHGB and TyG beyond the base model assessed by net reclassification improvement (NRI) and integrated discrimination improvement (IDI).

Comparison	Metric	Est.(95%CI)	*P*-value
Model 3 vs Model 3+TyHGB	IDI	0.003 (0.0007, 0.0063)	<0.0001
	NRI	0.2157 (0.132, 0.2694)	<0.0001
Model 3 vs Model 3+TyG	IDI	0.002 (0.0004, 0.0048)	0.002
	NRI	0.1433 (0.0796, 0.2061)	<0.0001
Model 3+TyG vs Model 3+TyHGB	IDI	0.001 (-0.0005, 0.003)	0.216
	NRI	0.0431 (-0.0636, 0.1314)	0.466

Model 3: adjusted for age, sex, current smoke, alcohol consumption, waist circumference, systolic blood pressure, family history of stroke and heart rate.

### Subgroup and interaction analyses

**Figure 4:**
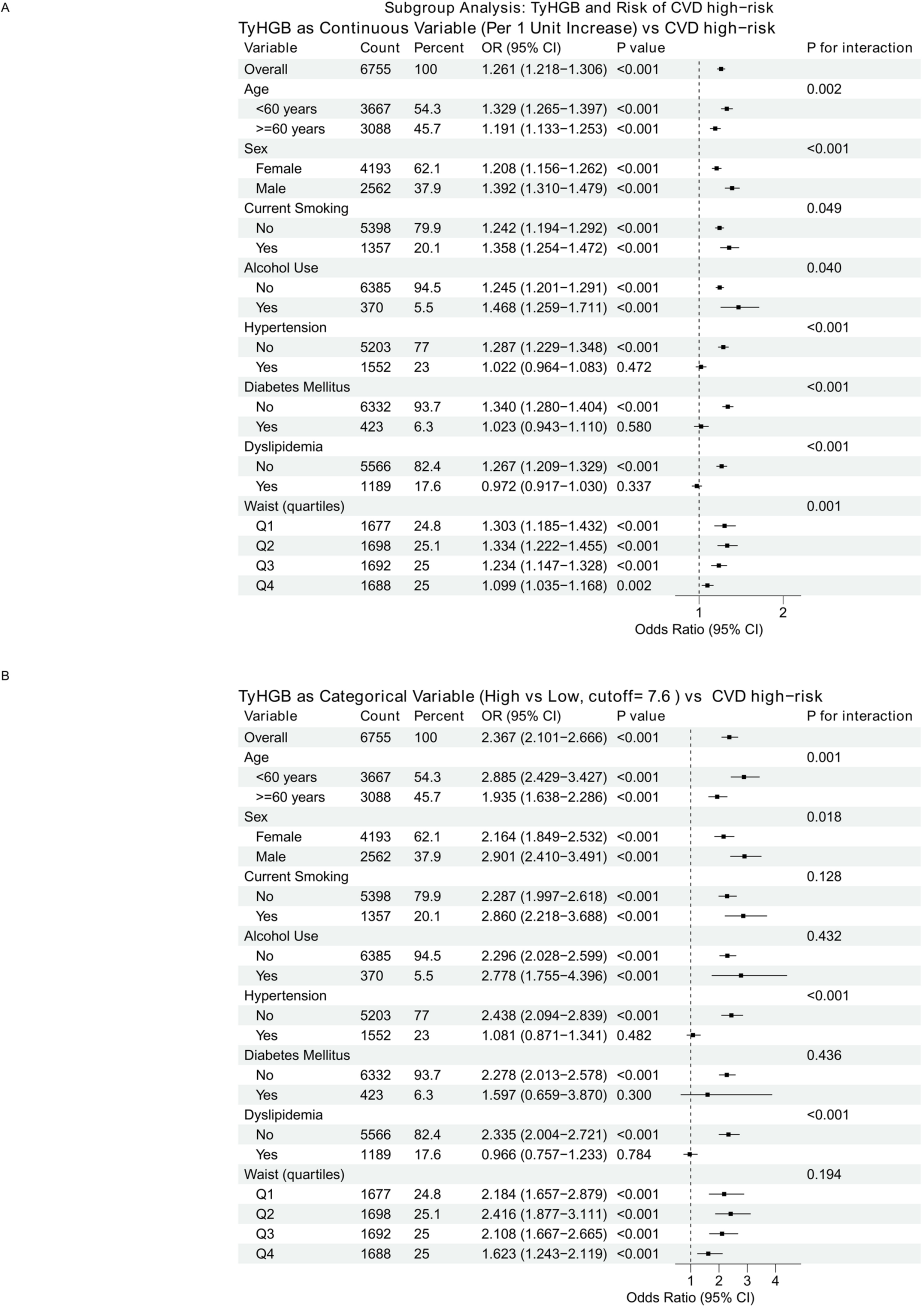
Subgroup analyses of the association between TyHGB and WHO-defined CVD high-risk status. Forest plots present ORs (95% CIs) across prespecified subgroups. **(A)** TyHGB modeled as a continuous variable (per 1-unit increase). **(B)** TyHGB modeled as a categorical variable (high vs low; cutoff =7.6). Subgroup-specific ORs were estimated using logistic regression within each subgroup (unadjusted models), and interaction *P* values were obtained from likelihood ratio tests.

In subgroup analyses, the positive association between TyHGB and CVD high-risk status was broadly consistent across prespecified strata. For TyHGB modeled continuously (per 1-unit increase), significant interaction was observed for age, sex, current smoking, alcohol use, hypertension, diabetes mellitus, dyslipidemia, and waist circumference quartiles; these findings remained materially unchanged after Benjamini-Hochberg FDR adjustment. When TyHGB was analyzed categorically (high vs low; cutoff = 7.6), significant effect modification was observed for age, sex, hypertension, and dyslipidemia, whereas no statistically significant interaction was observed for smoking, alcohol use, diabetes, or waist quartiles; this pattern was unchanged after Benjamini-Hochberg FDR adjustment (**Supplementary Table 3**; **Figure 4**).

**Figure 5:**
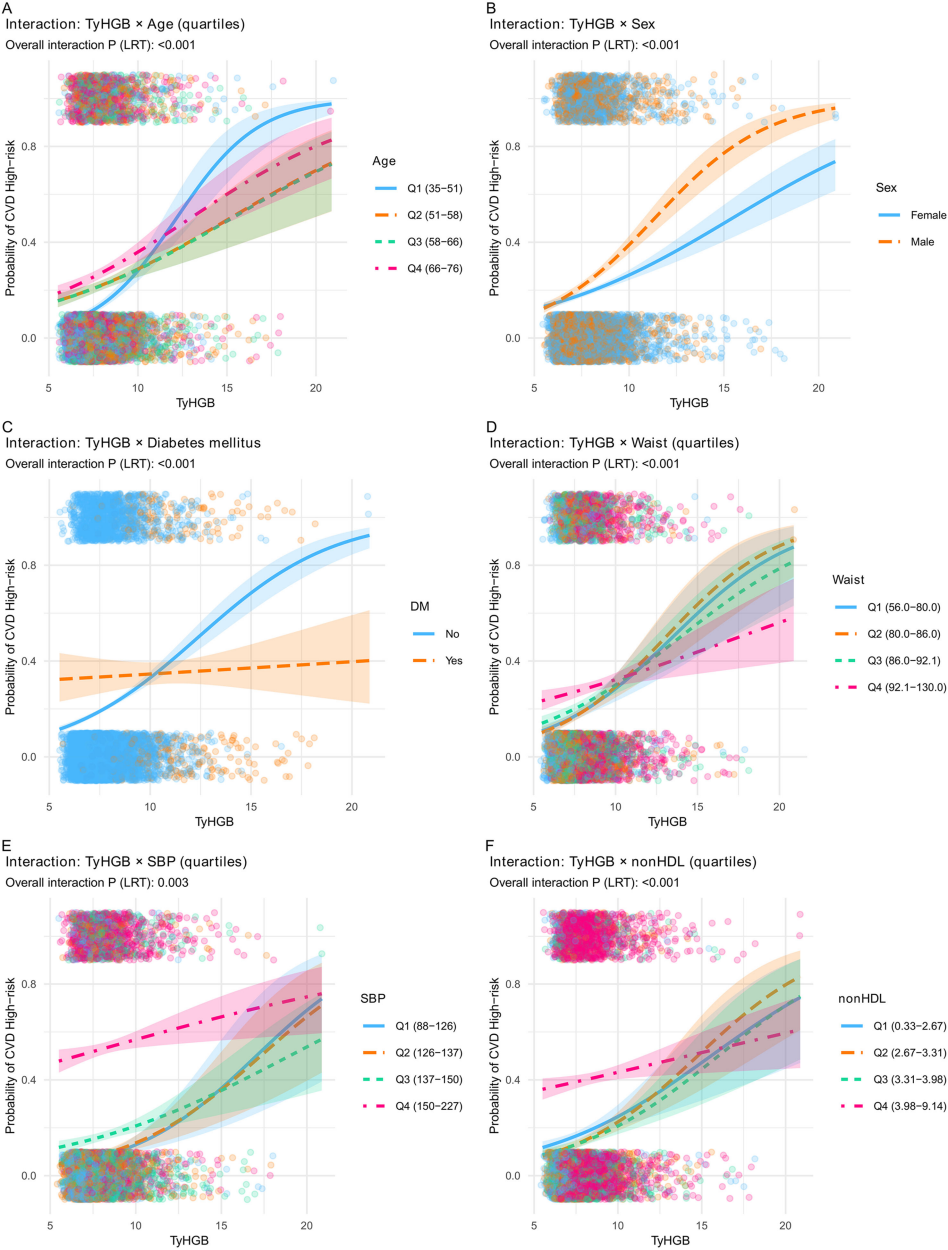
Interaction analyses showing heterogeneity in the TyHGB-CVD high-risk association across key strata. Predicted probabilities of WHO-defined CVD high-risk status are plotted across the TyHGB range, stratified by **(A)** sex, **(B)** age quartiles, **(C)** diabetes mellitus, **(D)** waist circumference quartiles, **(E)** non-HDL-C quartiles, and **(F)** systolic blood pressure (SBP) quartiles. Curves were derived from unadjusted logistic regression models including TyHGB, the moderator, and their interaction term. Overall interaction *P* values were obtained from likelihood ratio tests comparing models with and without the interaction.

Consistently, interaction curves showed significant TyHGB-by-stratum heterogeneity for age, sex, diabetes mellitus, waist circumference, SBP, and non-HDL-C (overall LRT *P* values <0.001 for most strata; SBP *P* = 0.003), indicating that the TyHGB–risk gradient differed across cardiometabolic backgrounds (**Figure 5**).

### Exploratory machine learning feature selection and interpretation (LASSO + RF + SHAP)

**Figure 6:**
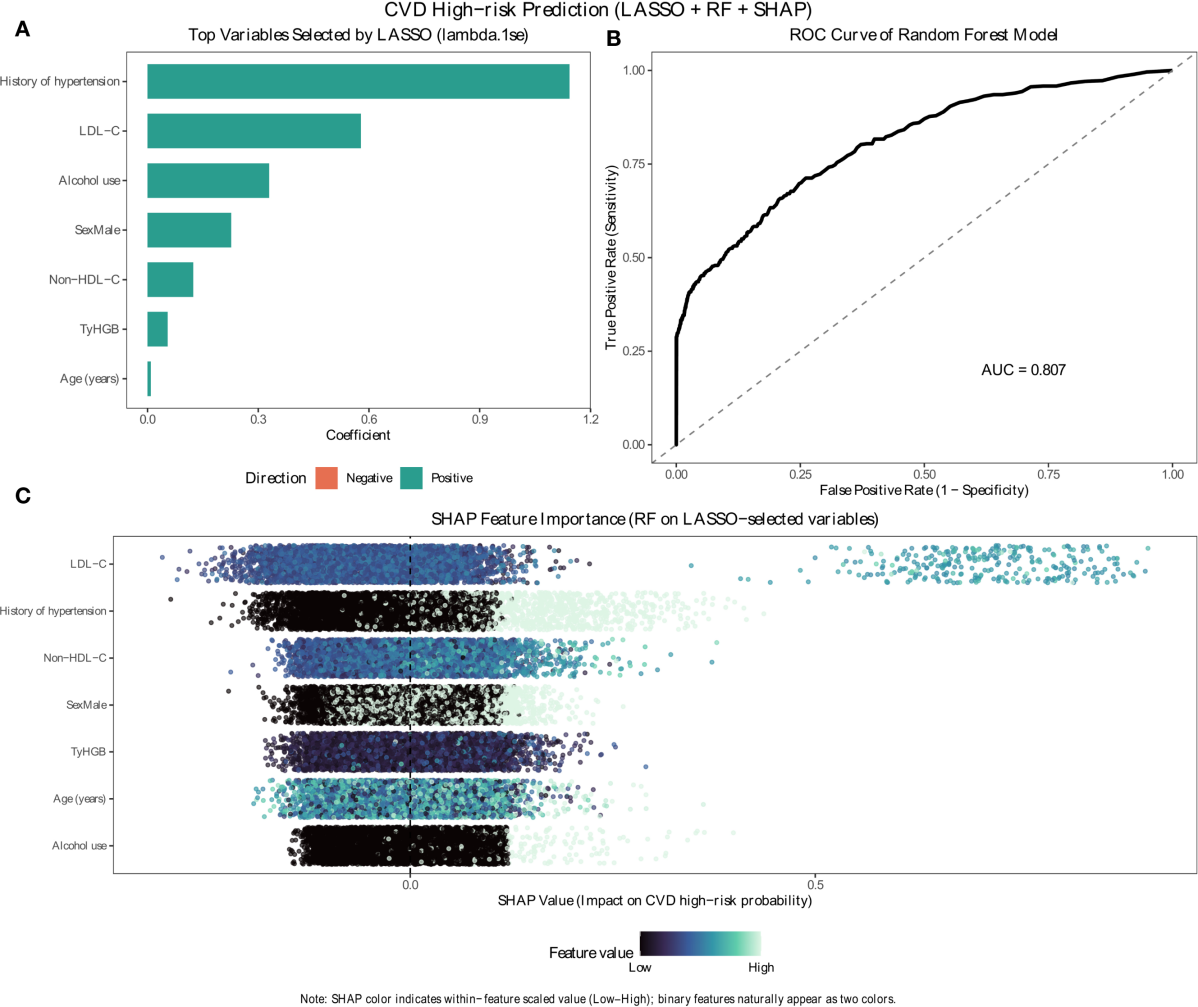
Exploratory machine learning analysis supporting the relative importance of TyHGB for WHO-defined CVD high-risk classification. **(A)** Predictors selected by LASSO at λ1se. **(B)** ROC curve of the random forest model trained on LASSO-selected predictors (AUC = 0.807). **(C)** SHAP summary plot for the random forest (color = feature value; positive SHAP indicates higher predicted CVD high-risk probability). In panel C, features are sorted by mean absolute SHAP value; thus, “rank x of y selected” denotes the feature’s importance rank among the y LASSO-selected predictors.

Within the pre-specified candidate set, LASSO selected seven variables with non-zero coefficients at λ1se: history of hypertension, LDL-C, alcohol use, male sex, non–HDL-C, TyHGB, and age (**Figure 6A**). The random forest model trained on these variables achieved an AUC of 0.807 on the test set (**Figure 6B**). In SHAP interpretation (**Figure 6C**), LDL-C (rank 1/7) and history of hypertension (rank 2/7) showed the greatest contribution, followed by non–HDL-C (rank 3/7) and male sex (rank 4/7); TyHGB ranked 5/7, and higher TyHGB values generally shifted predictions toward higher CVD high-risk probability (positive SHAP values) (**Figure 6C**).

## Discussion

In this community-based cross-sectional study derived from the ChinaHEART cohort, higher TyHGB was significantly associated with higher odds of CVD high-risk status defined by WHO CVD risk charts. Several complementary analyses supported the robustness and clinical relevance of this association: (1) TyHGB showed clear separation in distribution between CVD high-risk and non-high-risk groups; (2) TyHGB exhibited a significant (and nonlinear) dose–response relationship with CVD high-risk status; (3) TyHGB showed better discrimination than TyG in unadjusted comparisons, whereas the adjusted-model AUC differences were statistically significant but numerically small; and (4) TyHGB improved risk reclassification and discrimination beyond a conventional risk-factor model, as reflected by positive IDI and NRI.

### Principal findings

First, participants with high TyHGB had substantially higher prevalence of CVD high-risk status than those with low TyHGB (29% vs 15%; absolute difference, 14 percentage points). This pattern was accompanied by a less favorable cardiometabolic profile in the high TyHGB group, including higher waist circumference, blood pressure, triglycerides, and fasting glucose, and lower HDL-C, consistent with a clustered metabolic-risk phenotype. Accordingly, the odds ratios reported in the regression models should be interpreted as measures of association rather than direct absolute risk increments.

Second, in fully adjusted models controlling for major demographic, lifestyle, and hemodynamic risk factors (Model 3), TyHGB remained independently associated with CVD high-risk status (OR 1.131 per 1-unit increase), and categorically high TyHGB (≥7.6) showed an approximately 64% higher odds compared with low TyHGB. These findings suggest that the association of TyHGB with WHO-defined CVD high-risk status was not fully explained by the selected covariates in the main model; however, given the chart-derived nature of the outcome and the related cardiometabolic domains represented by several adjusted covariates, the adjusted estimates should be interpreted cautiously.

Third, RCS analyses suggested a significant nonlinear association for TyHGB, indicating that the risk gradient may not increase uniformly across the TyHGB range. In contrast, TyG displayed weaker evidence of nonlinearity (borderline/non-significant), implying that TyHGB might better reflect complex metabolic-risk accumulation across distributions.

### Sensitivity analysis and comparative performance with TyG

TyG was used as a sensitivity-analysis comparator because it is a widely recognized surrogate for insulin resistance and shares key components (TG and FPG) with TyHGB (5–10). TyG remained significantly associated with CVD high-risk status across models, supporting the overall biological plausibility that glyco-lipid dysregulation relates to high estimated CVD risk (8, 22, 23). TyHGB showed better discrimination than TyG in the unadjusted comparison (0.624 vs 0.585; DeLong *P* < 0.001), which represents the more substantive between-index difference observed in this study. By contrast, although AUC differences remained statistically significant in adjusted models, the absolute separation was minimal (for example, 0.818 vs 0.816 in Model 3) and should not be overinterpreted as a clinically meaningful superiority of TyHGB over TyG after conventional covariates were incorporated. This pattern is directionally consistent with emerging evidence from other metabolic-disease settings, in which TyHGB also showed stronger discriminatory performance than TyG (24), and with a recent report by Di Marco et al. showing that TG/HDL-C, but not TyG, was associated with arterial stiffness in individuals with prediabetes, which provides additional biological context for the inclusion of the TG/HDL-C component in TyHGB (25).

Beyond discrimination, reclassification analyses further suggested added utility. Adding TyHGB to the base model yielded greater improvements in IDI and NRI than adding TyG, whereas directly comparing TyHGB vs TyG on top of the base model did not show statistically significant differences in IDI/NRI—indicating that while TyHGB may provide stronger incremental signal, both indices share overlapping information when conventional covariates are already included. Thus, the potential added value of TyHGB may lie more in modest reclassification improvement than in a clinically meaningful gain in adjusted AUC over TyG. Importantly, the overall discrimination pattern remained directionally similar in overlap-excluded sensitivity analyses, and complementary analyses using self-reported history of CVD as a non-chart-derived prevalent outcome also provided supportive evidence, although these supplementary findings should be interpreted cautiously.

A key point in interpreting our findings is that the study outcome was WHO-defined CVD high-risk status, that is, an estimated 10-year risk category derived from a validated risk-chart framework, rather than adjudicated incident cardiovascular events. Accordingly, the observed associations and discrimination metrics should be interpreted as reflecting how TyHGB relates to cross-sectional classification into a higher estimated-risk group, not as evidence that TyHGB independently predicts future CVD events or lies on a confirmed causal pathway to those events. Because the chart-based outcome is constructed from established cardiometabolic domains, part of the observed discriminatory signal may reflect alignment between TyHGB and the broader metabolic-risk profile represented in the risk charts, rather than wholly independent prognostic information. For this reason, our results are best viewed as hypothesis-generating and as supporting the potential utility of TyHGB for risk stratification within a cross-sectional screening context.

### Subgroup and interaction findings

Among the observed interaction patterns, the sex difference was one of the most notable findings. The steeper TyHGB–CVD high-risk gradient in men may reflect known sexual dimorphism in adipose tissue distribution and metabolic regulation: compared with women, men generally accumulate more visceral fat and tend to exhibit less favorable tissue-specific insulin sensitivity and lipid handling at a similar level of adiposity. These differences may amplify the cardiometabolic consequences of a given degree of glyco-lipid dysregulation, making the TyHGB-associated gradient more apparent in men (26). Beyond sex, the observed effect modification by age is also biologically plausible, as adipose tissue distribution, substrate handling across adipose tissue-muscle-liver axes, and tissue-specific insulin sensitivity change with ageing, which may translate into heterogeneous cardiometabolic risk responses to a given level of glyco-lipid dysregulation and adiposity burden (26). Interestingly, the TyHGB-risk association showed heterogeneity across waist circumference quartiles, with a steeper gradient in the lower-to-mid quartiles. This may suggest that TyHGB captures adverse glyco-lipid dysregulation and insulin-resistance-related risk even among individuals with smaller waist circumference, where traditional anthropometric markers may underestimate cardiometabolic risk; conversely, in those with larger waist circumference, a higher baseline risk burden may attenuate the relative risk gradient in a cross-sectional setting; dysfunctional adipose tissue-endothelial crosstalk may amplify vascular injury under an insulin-resistant milieu and the interaction with diabetes mellitus may indicate that TyHGB provides greater risk stratification among individuals without diagnosed diabetes, potentially capturing earlier glyco-lipid dysregulation before overt diabetes and intensive treatment-related risk-factor modification (27). Moreover, modification by SBP is compatible with the bidirectional relationship between hypertension-related hemodynamic stress and endothelial dysfunction, which may potentiate downstream vascular damage when metabolic disturbances coexist. Finally, the interaction with non-HDL-C supports the notion that a higher atherogenic lipoprotein burden (captured by non-HDL-C/apoB-containing particles) could synergize with metabolic dysregulation, resulting in a steeper risk gradient across TyHGB levels in those with more pronounced atherogenic profiles (12). Because multiple subgroup interaction tests were performed, these heterogeneity findings should be interpreted cautiously; notably, the main interaction pattern remained similar after FDR adjustment, and the categorical-analysis interactions for smoking, alcohol use, diabetes, and waist quartiles were non-significant both before and after adjustment.

### Potential mechanisms and interpretation

TyHGB integrates information related to glyco-lipid metabolism (TG/HDL-C and FPG) and adiposity burden (BMI). Conceptually, this composite may better capture the overall metabolic load that contributes to vascular dysfunction and atherosclerotic processes through pathways including endothelial dysfunction, oxidative stress, pro-inflammatory activation, and dyslipoproteinemia (12, 13, 27, 28). HDL-C may partly reflect anti-atherogenic capacity, although HDL quantity does not fully represent HDL functionality under metabolic stress (29, 30). Conceptually, this composite may better capture the “metabolic load” that influences vascular function and atherosclerotic processes through multiple pathways, including endothelial dysfunction, pro-inflammatory activation, oxidative stress, and dyslipoproteinemia (31). The stronger discrimination and nonlinear dose-response pattern observed for TyHGB may reflect compounded risk when multiple metabolic domains deteriorate concurrently.

### Strengths and limitations

This study has strengths including a community-based sample from a structured national screening initiative, standardized measurement procedures, and comprehensive analyses incorporating dose–response modeling, discrimination, reclassification, and interaction assessment. We additionally complemented conventional regression analyses with an exploratory LASSO-based feature selection, random forest modeling, and SHAP interpretation, which consistently identified TyHGB as an informative predictor.

Several limitations should be noted. First, the cross-sectional design prevents causal inference and cannot establish temporal directionality. Second, CVD high-risk status was defined using WHO risk charts rather than adjudicated hard outcomes; thus, our findings reflect cross-sectional associations with estimated risk status rather than incident events. We selected the WHO risk charts because they provide a standardized framework suitable for community-based risk stratification, but alternative tools for Chinese populations, including the China-PAR equations, are also available 32,33; accordingly, the present findings should be interpreted specifically in relation to WHO-defined high-risk status rather than assumed to be identical across different cardiovascular risk algorithms (32, 33). This distinction is important because the chart-derived endpoint is not a direct measure of future observed cardiovascular disease, and therefore the present results should not be interpreted as demonstrating prospective predictive performance for hard CVD outcomes. In addition, because the chart-based classification is derived from established cardiometabolic domains, some of the observed association and discrimination may reflect conceptual proximity between TyHGB and the broader metabolic-risk profile represented in the risk charts, which complicates causal and mechanistic interpretation. To address this concern, we performed an overlap-excluded sensitivity ROC analysis by removing systolic blood pressure, waist circumference, and current smoking from the comparison framework; the overall direction of the discrimination findings remained similar, although absolute AUC values were attenuated (**Supplementary Figure 1A**). We also conducted a complementary ROC analysis using self-reported history of CVD, a non-chart-derived prevalent outcome; although the discrimination pattern remained supportive, this endpoint was retrospective and non-adjudicated and therefore cannot substitute for prospective hard cardiovascular outcomes (**Supplementary Figure 1B**). Third, although we adjusted for key confounders available in the dataset, residual confounding may remain (e.g., diet, physical activity intensity, socioeconomic factors). Although missingness was limited overall, incomplete data were present in several analysis variables, including TyHGB, TyG, and heart rate. Therefore, the complete-case analysis may still be susceptible to bias if the missing-data mechanism differed from the missing-at-random assumption. However, the MICE-based sensitivity analyses yielded nearly identical estimates, which reduces concern that exclusion of incomplete observations materially influenced the main findings. Moreover, because the outcome was defined by WHO risk charts, some candidate variables used in the exploratory machine learning workflow may overlap conceptually with chart inputs; therefore, LASSO/SHAP findings should be interpreted as supportive evidence rather than as causal or fully independent importance. In particular, stronger chart-adjacent predictors such as LDL-C and history of hypertension would be expected to rank ahead of TyHGB within the same model, so the lower SHAP rank of TyHGB should not be interpreted as absence of biological relevance, but rather as conditional importance in the presence of already-included strong predictors. Finally, the study was conducted in a regional branch of a national project, and external generalizability to other settings requires further validation.

### Implications

If confirmed in prospective studies, TyHGB-computed from routinely accessible metabolic and lipid measures-may offer a pragmatic adjunct for identifying individuals with elevated estimated CVD risk, particularly in primary care and community screening contexts. From a feasibility perspective, TyHGB requires only triglycerides, HDL-C, fasting glucose, and BMI, all of which are routinely available in standard fasting laboratory testing and basic anthropometric assessment; thus, the barrier to implementation would likely be low in settings where these measurements are already collected. Its incremental value beyond conventional covariates suggests potential usefulness for refining prevention prioritization and guiding early lifestyle or pharmacologic interventions.

## Conclusion

In a community-based ChinaHEART cohort sample, higher TyHGB was independently associated with WHO-defined CVD high-risk status. TyHGB demonstrated a significant nonlinear dose-response relationship, better discrimination than TyG in unadjusted analyses, and modest incremental reclassification and discrimination beyond a conventional risk-factor model. These findings should be interpreted as relating to estimated risk status rather than adjudicated incident CVD events. Prospective studies with adjudicated hard cardiovascular endpoints (e.g., major adverse cardiovascular events), adequate follow-up duration (preferably at least 5 years), and validation across both community-based and external comparison populations are warranted to determine whether TyHGB provides independent prognostic information and can be developed into a practical screening adjunct.

## Data Availability

The data that support the findings of this study are available from the corresponding author Haoran Wang (d201278406@alumni.hust.edu.cn), upon reasonable request. Requests to access these datasets should be directed to Haoran Wang, d201278406@alumni.hust.edu.cn.

## Supplementary material

Supplementary Figure 1 Supplementary ROC analyses for overlap-excluded sensitivity testing and complementary evaluation using self-reported history of CVD. **(A)** Overlap-excluded sensitivity ROC analysis comparing the full Model 3 plus TyHGB, the reduced Model 3 excluding systolic blood pressure, waist circumference, and current smoking plus TyHGB, the same reduced model plus TyG, and the reduced base model without either index. **(B)** Complementary ROC analysis of TyHGB for self-reported history of cardiovascular disease under Model 1 (crude), Model 2 (adjusted for age and sex), and Model 3 (fully adjusted). AUCs are presented with bootstrap 95% confidence intervals, and paired DeLong tests were used for model comparisons on the same analytic sample.

## Glossary

AUC: Area under the curve
BMI: Body mass index
CI: Confidence interval
CVD: Cardiovascular disease
DBP: Diastolic blood pressure
FPG: Fasting plasma glucose
HDL-C: High-density lipoprotein cholesterol
IDI: Integrated discrimination improvement
IR: Insulin resistance
LRT: Likelihood ratio test
LASSO: Least absolute shrinkage and selection operator
NRI: Net reclassification improvement
OR: Odds ratio
RF: Random forest
RCS: Restricted cubic spline
ROC: Receiver operating characteristic
SBP: Systolic blood pressure
SHAP: Shapley additive explanations
STROBE: Strengthening the Reporting of Observational Studies in Epidemiology
TG: Triglycerides
TyG: Triglyceride-glucose index
TyHGB: Triglyceride-to-HDL-C ratio, fasting plasma glucose, and body mass index composite index
WHO: World Health Organization
