# Supplementary figures and images for: TyHGB and CVD high-risk stratification: nonlinear association and discrimination in the ChinaHEART Luohe study
Source: Front Endocrinol (Lausanne). 2026 May 7;17:1818472. doi: 10.3389/fendo.2026.1818472 (PMC13189912; doi:10.3389/fendo.2026.1818472)

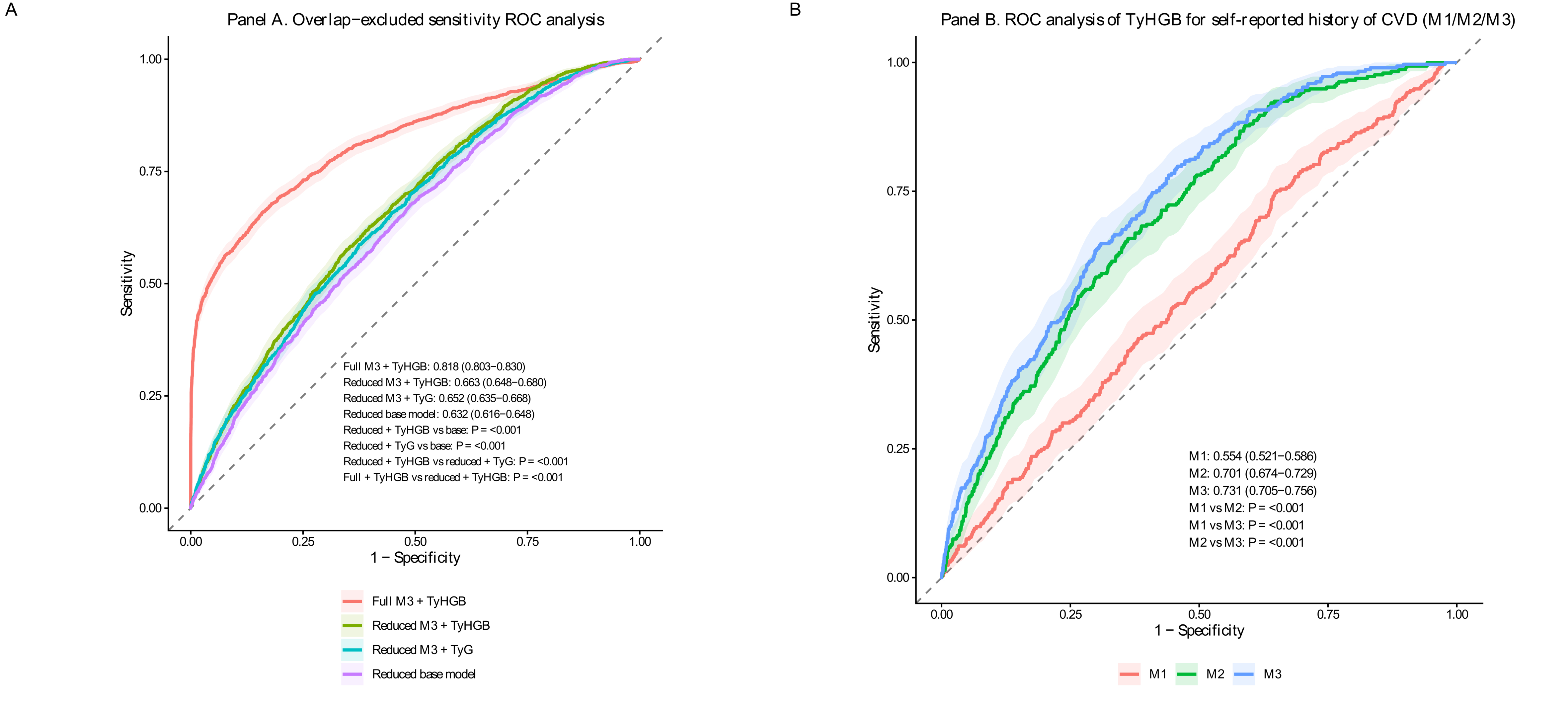

Supplement: Supplementary Figure 1 — Supplementary ROC analyses for overlap-excluded sensitivity testing and complementary evaluation using self-reported history of CVD. (A) Overlap-excluded sensitivity ROC analysis comparing the full Model 3 plus TyHGB, the reduced Model 3 excluding systolic blood pressure, waist circumference, and current smoking plus TyHGB, the same reduced model plus TyG, and the reduced base model without either index. (B) Complementary ROC analysis of TyHGB for self-reported history of cardiovascular disease under Model 1 (crude), Model 2 (adjusted for age and sex), and Model 3 (fully adjusted). AUCs are presented with bootstrap 95% confidence intervals, and paired DeLong tests were used for model comparisons on the same analytic sample. [file Image1.jpeg]
