# Supplementary material for: TyHGB and CVD high-risk stratification: nonlinear association and discrimination in the ChinaHEART Luohe study
Source: Front Endocrinol (Lausanne). 2026 May 7;17:1818472. doi: 10.3389/fendo.2026.1818472 (PMC13189912; doi:10.3389/fendo.2026.1818472)
Supplement: Supplementary file 2 [file Table1.docx]

Supplementary Table S1. Adjusted absolute differences (percentage-point differences) in WHO-defined CVD high-risk classification derived from the same logistic models as Table 2

| Exposure | Model | Contrast | N | Adjusted absolute difference (percentage points) |
| --- | --- | --- | --- | --- |
| TyHGB (per 1-unit increase) | Model 1 | Per 1-unit increase | 6,750 | 4.1 (3.4, 4.7) |
| TyHGB (per 1-unit increase) | Model 2 | Per 1-unit increase | 6,750 | 4.0 (3.4, 4.7) |
| TyHGB (per 1-unit increase) | Model 3 | Per 1-unit increase | 6,750 | 1.6 (0.9, 2.2) |
| High TyHGB (≥7.6) vs Low TyHGB (<7.6) | Model 1 | High vs Low | 6,750 | 14.4 (12.5, 16.3) |
| High TyHGB (≥7.6) vs Low TyHGB (<7.6) | Model 2 | High vs Low | 6,750 | 14.3 (12.5, 16.2) |
| High TyHGB (≥7.6) vs Low TyHGB (<7.6) | Model 3 | High vs Low | 6,750 | 6.2 (4.3, 8.2) |
| TyG (per 1-unit increase) | Model 1 | Per 1-unit increase | 6,750 | 11.1 (8.7, 13.5) |
| TyG (per 1-unit increase) | Model 2 | Per 1-unit increase | 6,750 | 12.3 (9.8, 14.7) |
| TyG (per 1-unit increase) | Model 3 | Per 1-unit increase | 6,750 | 3.8 (2.1, 5.8) |
