# Supplementary material for: TyHGB and CVD high-risk stratification: nonlinear association and discrimination in the ChinaHEART Luohe study
Source: Front Endocrinol (Lausanne). 2026 May 7;17:1818472. doi: 10.3389/fendo.2026.1818472 (PMC13189912; doi:10.3389/fendo.2026.1818472)
Supplement: Supplementary file 3 [file Table2.docx]

## *Supplement Table S2. Sensitivity analysis: shared complete-case (CC) versus mutiple imputation by chained equation (MICE)-based logistic regression*

### A. Sample-size summary

| **Metric** | **Value** |
| --- | --- |
| Total enrolled participants | 6,860 |
| Participants with observed outcome | 6,860 |
| Shared complete-case regression dataset | 6,750 |
| Excluded from shared complete-case dataset | 110 |
| Number of imputations (m) | 20 |
| Maximum iterations | 20 |

### B. Regression results comparison

| **Exposure** | **Model** | **N_CC** | **CC_OR_95CI** | **CC_*P*** | **N_MICE** | **MICE_OR_95CI** | **MICE_*P*** |
| --- | --- | --- | --- | --- | --- | --- | --- |
| TyHGB (per 1-unit increase) | Model 1 | 6,750 | 1.260 (1.217, 1.306) | <0.001 | 6,860 | 1.265 (1.222, 1.310) | <0.001 |
| TyHGB (per 1-unit increase) | Model 2 | 6,750 | 1.263 (1.219, 1.309) | <0.001 | 6,860 | 1.269 (1.225, 1.315) | <0.001 |
| TyHGB (per 1-unit increase) | Model 3 | 6,750 | 1.131 (1.081, 1.183) | <0.001 | 6,860 | 1.134 (1.084, 1.186) | <0.001 |
| High TyHGB (≥7.6) vs Low TyHGB (<7.6) | Model 1 | 6,750 | 2.359 (2.094, 2.658) | <0.001 | 6,860 | 2.381 (2.115, 2.682) | <0.001 |
| High TyHGB (≥7.6) vs Low TyHGB (<7.6) | Model 2 | 6,750 | 2.398 (2.124, 2.707) | <0.001 | 6,860 | 2.426 (2.150, 2.738) | <0.001 |
| High TyHGB (≥7.6) vs Low TyHGB (<7.6) | Model 3 | 6,750 | 1.642 (1.408, 1.916) | <0.001 | 6,860 | 1.652 (1.416, 1.927) | <0.001 |
| TyG (per 1-unit increase) | Model 1 | 6,750 | 1.777 (1.591, 1.984) | <0.001 | 6,860 | 1.799 (1.612, 2.007) | <0.001 |
| TyG (per 1-unit increase) | Model 2 | 6,750 | 1.915 (1.708, 2.147) | <0.001 | 6,860 | 1.947 (1.738, 2.182) | <0.001 |
| TyG (per 1-unit increase) | Model 3 | 6,750 | 1.343 (1.171, 1.540) | <0.001 | 6,860 | 1.361 (1.188, 1.559) | <0.001 |

### C. MICE methods used

| **Variable** | **Missing** | **Method** |
| --- | --- | --- |
| CVD risk | 0 | NA |
| TyHGB ;TyG | 57 | Predictive mean matching |
| TyHGB | 48 | Predictive mean matching |
| age | 0 | NA |
| sex | 0 | NA |
| smoking | 0 | NA |
| Alcohol consumption | 0 | NA |
| Systolic blood pressure | 0 | NA |
| Waist circumference | 0 | NA |
| Family history of stroke | 0 | NA |
| Heart rate | 5 | Predictive mean matching |

Notes: The complete-case (CC) analysis used the same shared regression analytic dataset as the original regression code, requiring complete data for CVD risk, TyHGB, TyG, and the prespecified covariates (age, sex, smoking, alcohol consumption, systolic blood pressure, waist circumference, family history of stroke, heart rate). For the multiple imputation by chained equation (MICE) analysis, participants with observed outcome data were retained, and missing predictor values were imputed under a missing-at-random assumption. The outcome was not imputed. TyHGB cat was derived after imputation within each imputed dataset. Pooled MI estimates were obtained using Rubin's rules.
