# Supplementary material for: TyHGB and CVD high-risk stratification: nonlinear association and discrimination in the ChinaHEART Luohe study
Source: Front Endocrinol (Lausanne). 2026 May 7;17:1818472. doi: 10.3389/fendo.2026.1818472 (PMC13189912; doi:10.3389/fendo.2026.1818472)
Supplement: Supplementary file 4 [file Table3.docx]

## *Supplementary Table S3. Benjamini-Hochberg FDR-adjusted interaction P-values for prespecified subgroup analyses of TyHGB and WHO-defined CVD high-risk status*

| **Subgroup** | **Levels** | **N** | **Continuous TyHGB**  **LRT df** | **Continuous TyHGB**  **Raw P for interaction** | **Continuous TyHGB**  **BH-FDR adjusted P** | **Continuous TyHGB**  **FDR <0.05** | **Categorical TyHGB**  **LRT df** | **Categorical TyHGB**  **Raw P for interaction** | **Categorical TyHGB**  **BH-FDR adjusted P** | **Categorical TyHGB**  **FDR <0.05** |
| --- | --- | --- | --- | --- | --- | --- | --- | --- | --- | --- |
| Age | <60 years (n=3667)  ≥60 years (n=3088) | 6,755 | 1 | 0.002 | 0.003 | Yes | 1 | 0.001 | 0.003 | Yes |
| Sex | Female (n=4193)  Male (n=2562) | 6,755 | 1 | <0.001 | <0.001 | Yes | 1 | 0.018 | 0.036 | Yes |
| Current smoking | No (n=5398)  Yes (n=1357) | 6,755 | 1 | 0.049 | 0.048 | Yes | 1 | 0.128 | 0.203 | No |
| Alcohol use | No (n=6385)  Yes (n=370) | 6,755 | 1 | 0.040 | 0.040 | Yes | 1 | 0.432 | 0.448 | No |
| Hypertension | No (n=5203)  Yes (n=1552) | 6,755 | 1 | <0.001 | <0.001 | Yes | 1 | <0.001 | <0.001 | Yes |
| Diabetes mellitus | No (n=6332)  Yes (n=423) | 6,755 | 1 | <0.001 | <0.001 | Yes | 1 | 0.436 | 0.448 | No |
| Dyslipidemia | No (n=5566)  Yes (n=1189) | 6,755 | 1 | <0.001 | <0.001 | Yes | 1 | <0.001 | <0.001 | Yes |
| Waist circumference quartiles | Q1 (n=1677)  Q2 (n=1698)  Q3 (n=1692)  Q4 (n=1688) | 6,755 | 3 | 0.001 | 0.001 | Yes | 3 | 0.194 | 0.259 | No |

Abbreviations: LRT, likelihood ratio test; FDR, false discovery rate.

Notes: Raw interaction P values were obtained from likelihood ratio tests comparing unadjusted logistic models with and without the interaction term. Benjamini-Hochberg false discovery rate correction was applied separately within the continuous TyHGB interaction family and the categorical TyHGB interaction family (8 tests in each family). Categorical TyHGB was defined using the ROC-derived cutoff of 7.6; high vs low. Subgroup definitions were identical to those used in the primary subgroup forest plot.
